# Human cells with osteogenic potential in bone tissue research

**DOI:** 10.1186/s12938-023-01096-w

**Published:** 2023-04-03

**Authors:** Jana Dvorakova, Lucie Wiesnerova, Petra Chocholata, Vlastimil Kulda, Lukas Landsmann, Miroslava Cedikova, Michaela Kripnerova, Lada Eberlova, Vaclav Babuska

**Affiliations:** 1 Department of Medical Chemistry and Biochemistry, Faculty of Medicine in Pilsen, Charles University, Alej Svobody 1655/76, 323 00 Plzen, Czech Republic; 2 Biomedical Center, Laboratory of Tumor Biology and Immunotherapy, Faculty of Medicine in Pilsen, Charles University, Alej Svobody 1655/76, 323 00 Plzen, Czech Republic; 3 Department of Biology, Faculty of Medicine in Pilsen, Charles University, Alej Svobody 1655/76, 323 00 Plzen, Czech Republic; 4 Department of Anatomy, Faculty of Medicine in Pilsen, Charles University, Alej Svobody 1655/76, 323 00 Plzen, Czech Republic

**Keywords:** Bone engineering, Osteosarcoma cell lines, Mesenchymal stem cells, Human osteoblasts, Osteodifferentiation

## Abstract

Bone regeneration after injury or after surgical bone removal due to disease is a serious medical challenge. A variety of materials are being tested to replace a missing bone or tooth. Regeneration requires cells capable of proliferation and differentiation in bone tissue. Although there are many possible human cell types available for use as a model for each phase of this process, no cell type is ideal for each phase. Osteosarcoma cells are preferred for initial adhesion assays due to their easy cultivation and fast proliferation, but they are not suitable for subsequent differentiation testing due to their cancer origin and genetic differences from normal bone tissue. Mesenchymal stem cells are more suitable for biocompatibility testing, because they mimic natural conditions in healthy bone, but they proliferate more slowly, soon undergo senescence, and some subpopulations may exhibit weak osteodifferentiation. Primary human osteoblasts provide relevant results in evaluating the effect of biomaterials on cellular activity; however, their resources are limited for the same reasons, like for mesenchymal stem cells. This review article provides an overview of cell models for biocompatibility testing of materials used in bone tissue research.

## Introduction

Bone diseases are serious medical problems that are a major financial burden on healthcare. Worldwide, bone graft transplantation is the second most commonly performed tissue transplant after blood [1]. Intensive research is currently being conducted in the field of bone tissue engineering to regenerate tissues damaged by injury or disease. The findings of this research can be applied in the area of dentistry, such as dental implantology, maxillofacial surgery or periodontology (e.g., alveolar ridge remodeling, treatment of bone defects around dental implants, or filling bone defects after extraction). Another area of application is orthopedics and traumatology (traumatic bone defects and pathological fractures due to bone cysts, ganglia and tumors) [2].

Under physiological conditions, bone is constantly being remodeled through the activity of osteoblasts and osteoclasts, so that healthy bone tissue is able to naturally repair significant injuries [3]. If the tissue damage exceeds bone reparative capacity, external intervention is required. The ideal for medical use is autologous tissue, also called an autograft (most often taken from the ridge of the hip bone), which eliminates the immunogenic response and is optimal for cell growth. However, autograft availability is often limited by the patient’s comorbidities; alternatively, there may not be enough source material due to the limited number of surgical sites [4]. The next option is to use grafts from other donors (allografts), but in this case there is a risk about the immunoreactions and transmission of infections. For the above reasons, the use of artificial materials is a good alternative [5].

Various materials to replace the missing bone tissue are being tested, but only a few of them have shown satisfactory results [6]. Both inorganic and organic materials, based on natural and synthetic polymers, are used in bone tissue engineering. Some scaffolds combine several materials to maximize their beneficial properties [7]. In addition, the surfaces of the materials can be treated with various chemical or physico-chemical methods to protect and promote tissue healing [8].

Biomaterials are selected not only for their mechanical properties, but also for their optimal behavior in the physiological environment (biocompatibility), the ability of a material to induce an appropriate response in a recipient in a specific situation. The biocompatible material should be non-toxic, non-allergenic and non-carcinogenic [9] and it should also promote cell proliferation, differentiation, and functional tissue development [10]. A scaffold or an implant manufactured for bone support and substitution must have the key osteobiological properties: osteoconductivity (the ability for cellular communication across and through the substrate), osteoinductivity (the material is able to stimulate the differentiation of cells towards the osteogenic lineage), and osseointegration (the material is able to physically and functionally integrate with the living tissue) [11]. For some materials, biodegradability is also required. Biodegradability is suitable for materials used for temporary tissue replacement serving at the same time as scaffolds promoting tissue regeneration [12]. On the other hand, degradability is undesirable for materials serving as permanent tissue substitution, such as titanium implants [13].

Various human and animal cell cultures are used for in vitro testing of material biocompatibility. Most often, these are malignant cell lines that can be commercially available, mesenchymal stem cells that can be taken from the organism, or primary cells directly obtained from the living tissue. As we discuss below, the use of any cell model has its advantages and disadvantages that should be considered. The aim of this article is to provide an overview of cell models for testing the biocompatibility of materials used to replace hard tissues, which should help to select the appropriate cells for a particular application. The most commonly used methods for evaluation of cell morphology, adhesion, proliferation and osteodifferentiation are also summarized.

## Bone microenvironment and hormonal regulation of bone

Bone is a complex, plastic organ that supports the body, protects soft organs, and also performs endocrine functions [14]. In mature bone, about 40% of the dry weight consists of organic components—osteoid (type I collagen, glycosaminoglycans, and glycoproteins, such as osteopontin and osteocalcin); the rest is formed by inorganic salts, such as hydroxyapatite, calcium phosphate, and calcium carbonate [15].

In macroscopic appearance, we distinguish between spongy and compact bone in mature bone tissue. The compact (cortical or dense) bone forms the outer shell of each bone. It is formed by the osteons (Haversian systems)—cylindrical units formed by 4–20 lamellae around the central (Haversian) canal, through which the vascular and nervous supplies pass. Between the lamellae, the central vessels interconnect via transverse Volkmann canals. The spongy (cancellous, trabecular) bone lacks the regular lamellar osteons. It forms a network of plate-like trabeculae surrounded by connective tissue and bone marrow. Spongy bone has a larger surface area and a higher metabolic rate than compact bone [16]. Except for the articular surfaces, compact bone is covered by highly vascularized and innervated periosteum on the outside, and endosteum on the inside [17].

The bone marrow, a hematopoietic organ consisting of a spatial network of reticular fibers, macrophages, and blood capillaries, is found within the central cavities of axial and long bones, and also fills the spaces within spongy bone. In this fibrous network, there are pluripotent stem cells, from which the progenitor cells for the myeloid and lymphoid lines are formed, as well as mesenchymal cells capable of differentiating into osteoblasts. In adulthood, active bone marrow persists in the flat bones of the skull, in the bones of axial skeleton (sternum, ribs), in the pelvic bone and also in the appendicular long bones [18].

**Fig. 1:**
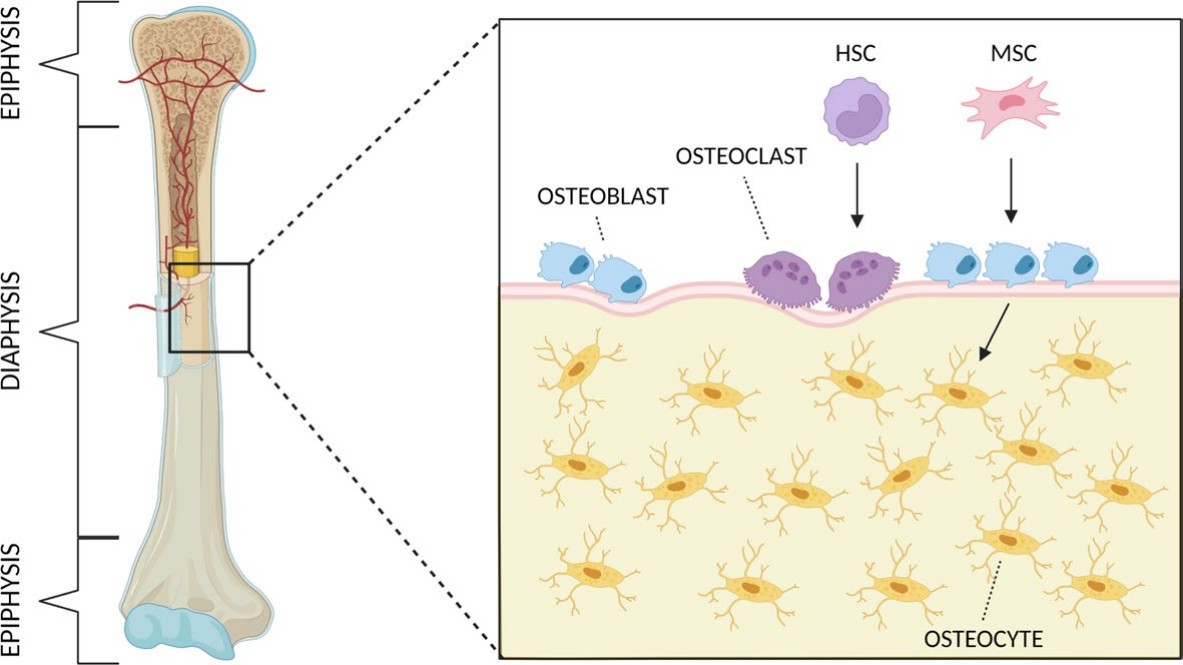
Schematic representation of bone structure. Osteoblasts derive from mesenchymal stem cells (MSC) and their main function is bone formation. Osteocytes, the most numerous type of cells in the bone, arise from osteoblasts and function in response to mechanical stress and regulation of local and systemic mineral homeostasis. Osteoclasts, which derive from hematopoietic stem cells (HSC), function in bone resorption. Created with BioRender.com

The bone microenvironment includes a number of stem cells of mesenchymal origin, which allow the bone to grow and support a strong ability to heal and regenerate after damage [19]. The periosteum and endosteum are the sources of mesenchymal osteoprogenitor cells, from which osteoblasts are formed. Osteoblasts are responsible for new bone formation and produce unmineralized osteoid and alkaline phosphatase, a surface protein promoting mineralization. The most distinctive histological feature of the osteoblasts is their intense cytoplasmic basophilia. After being surrounded by osteoid, osteoblasts change their metabolic activity, reside in the lacunae, and become osteocytes. Osteocytes interconnect via tiny canaliculi and cytoplasmic protrusions that permit the transfer of molecules, nutrients, and hormones. Other cells present in bone tissue include osteoclasts, which are multinucleate cells formed by the fusion and differentiation of several monocytes. They are responsible for bone resorption [20]. A schematic representation of bone structure is shown in Fig. 1.

Bone tissue is regulated by a number of hormones that are critical for optimal skeletal growth and bone density [21]. Major hormonal regulators of the bone remodeling process include calcitriol, parathyroid hormone (PTH), calcitonin, estrogen, and androgens; glucocorticoids, thyroid hormone and growth hormone also influence bone remodeling [22–24]. Secretion of calcitriol, calcitonin, and PTH is driven by the plasma calcium level. Calcitriol, the most active metabolite of vitamin D, promotes the absorption of calcium in the intestine and its reabsorption from the glomerular filtrate in the kidneys. It is essential for bone development and health [25]. Calcitonin decreases plasma calcium by storing it in bone tissue and has the ability to inhibit osteoclast activity [26]. PTH maintains serum calcium homeostasis by increasing osteoclast activity and calcium reabsorption in the kidneys [27]. Growth hormone stimulates proliferation of osteoprogenitor cells during chondrogenic ossification [28]. Estrogens and androgens during pubescence accelerate bone growth and skeletal maturation [29].

## Osteogenic differentiation and its testing

### Osteogenic differentiation

There are several transcription factors that direct multipotent mesenchymal cells to an osteoblastic lineage. The key transcription factor initiating osteodifferentiation of the mesenchymal cell is runt-related transcription factor 2 (Runx2) [30].

After differentiation into preosteoblasts, Runx2, osterix and β-catenin direct the cells to become immature osteoblasts [31]. Osterix (Osx, Sp7), a zinc finger-containing osteoblast-specific transcription factor located in the nucleus, induces the expression of a number of genes involved in the mineralization process in immature and mature osteoblasts. It inhibits chondrocyte differentiation and maintains a balance between cartilage and bone differentiation [32]. β-Catenin is a central molecule of the multi-component Wnt signaling pathway, an important mediator of cell fate determination [33]. The β-catenin pathway undergoes crosstalk with the signaling of bone morphogenetic protein-2 (BMP2) produced by osteocytes and regulating anabolic functions of osteoblasts. These two pathways cooperatively regulate osteoblast differentiation and bone formation [34].

Immature osteoblasts produce many specific proteins, including alkaline phosphatase, collagen type I, osteopontin and bone sialoprotein and so block their potential to differentiate into the chondrocytic and adipocytic lineage [31, 35].

Human alkaline phosphatase (ALP) occurs at least in four tissue-specific isoforms, known as placental, intestinal, liver/bone/kidney and germ cell. ALP is a membrane-bound enzyme expressed in immature osteoblast cells. It is considered to be a marker for their early differentiation and plays a key role in the calcification of bones [35]. ALP hydrolyzes pyrophosphate and supplies inorganic phosphate to enhance mineralization as well as to reduce extracellular pyrophosphate, an inhibitor of mineral formation [36].

Collagen type I (Col1) is an essential building element of the extracellular matrix (ECM) in connective tissues, including bone. It binds to other ECM proteins and cell surface integrins. Col1 mediates cell adhesion, proliferation and differentiation of the osteoblast phenotype, and can, therefore, be considered as an early marker of osteodifferentiation [37]. Mutation in Col1 gene causes osteogenesis imperfecta [38].

Bone sialoprotein (BSP) is a non-collagenous protein playing an important role in ECM mineralization by calcium incorporation and nodule formation. Its other role is in facilitating the adhesion of osteoclasts to the bone surface. It can be used as a marker of osteoblast differentiation [39].

Osteopontin (OPN) is an adhesive glycophosphoprotein, which is present not only in the bone and teeth but also in many other tissues and body fluids, such as kidneys, epithelial tissues, blood plasma and breast milk [37], in which tissues it has many diverse functions [40]. In bone, OPN is released by osteoblasts and osteoclasts. It promotes the absorption and mineralization of the bone matrix and plays an important role in the process of bone formation and remodeling [41]. Together with BSP, OPN is necessary not only for the mineralization process, but also for the repair of mineralized tissue [40, 42]. OPN has been shown to play an important role in inflammation, biomineralization, cardiovascular disease, cell viability, cancer, diabetes and kidney stone disease through various mechanisms [42].

Osteonectin (SPARC, BM-40) is another non-collagenous protein occurring in mineralized tissues; its expression is closely aligned with Col1 expression. It is secreted by osteoblasts during bone formation. In the osteoid, osteonectin binds collagen and crystals of hydroxyapatite and releases calcium ions, possibly enhancing mineralization of the collagen matrix in bones. Osteonectin is highly expressed in active osteoblasts, bone marrow progenitor cells, odontoblasts, periodontal ligament fibroblasts, and hypertrophic chondrocytes; in mature bone its expression decreases [43].

In order for bone maturation to continue, Runx2 expression must be downregulated, because it inhibits osteoblast maturation by maintaining in the immature stage [30]. Consequently, ALP expression is reduced, while osteocalcin expression increases [36].

Osteocalcin (OCN) is the most abundant non-collagenous protein found in bones, and can serve as a suitable marker for osteogenic maturation [44]. It is considered to be a late indicator of osteodifferentiation and a terminal symbol of hard tissue regeneration [37]. Mature osteoblasts express high levels of OCN at the time they are finally embedded in the bone matrix to become osteocytes [31].

Osteocytes release fibroblast growth factors (FGFs), BMPs, receptor activator of nuclear factor κB ligand (RANKL), osteoprotegerin (OPG) and sclerostin (SOST). FGFs and BMPs regulate osteoblast activity. RANKL induces osteoclast differentiation [45]. OPG reduces osteoclastogenesis and protects the skeleton from excessive bone resorption by binding to RANKL and so preventing activation of the RANK receptor [46]. Research shows that the OPG–RANK–RANKL system modulates cancer cell migration, thus controlling the development of bone metastases [47]. The glycoprotein SOST is a negative regulator of bone mass due to the inhibition of bone formation by osteoblasts. Thus, osteocytes control bone resorption and deposition by controlling the activity of osteoclasts and osteoblasts [45].

**Fig. 2:**
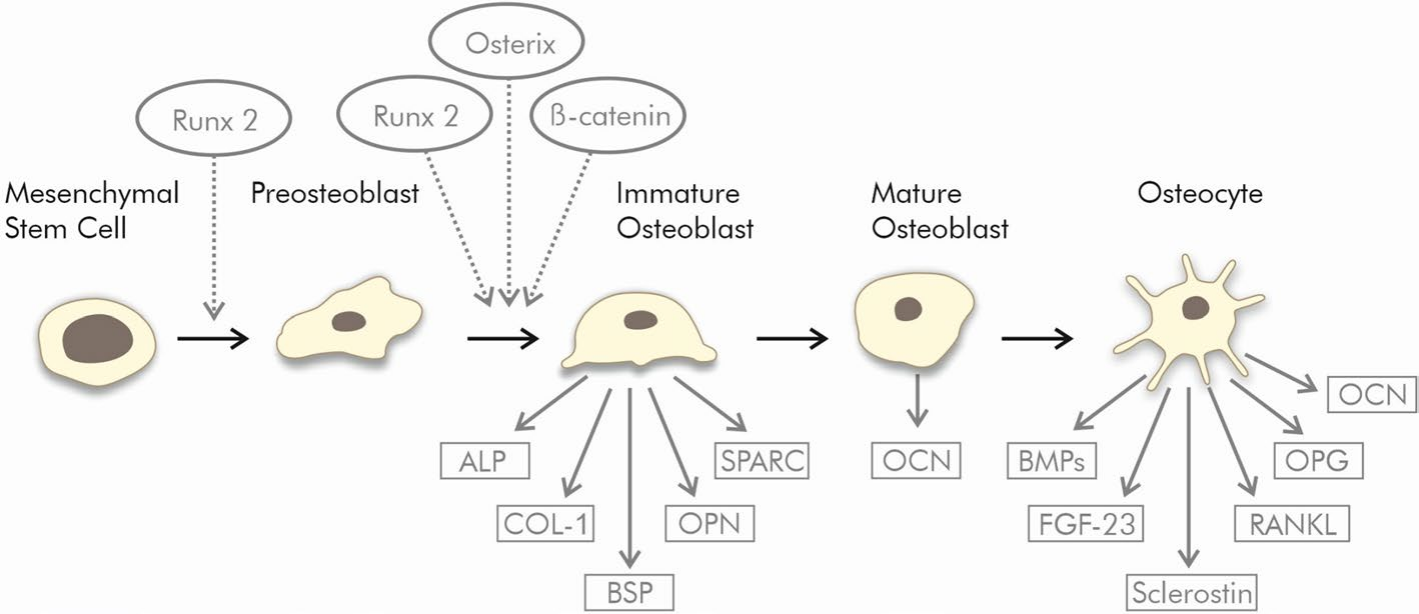
Osteogenic differentiation of the mesenchymal stem cells

The regulation of differentiation into osteoblasts and osteocytes from mesenchymal stem cells is shown in Fig. 2.

### Monitoring of cell morphology, adhesion and proliferation

The physico-chemical properties and surface modifications of materials used as bone prostheses and implants can affect cell adhesion, proliferation and morphogenesis [48]. Evaluation of the material’s influence on cell behavior can be performed by a variety of methods, some of which are briefly described here.

A crystal violet staining assay can be applied to analyze the basic approximate shape of fixed cells [49]. In our experience, either 4% formaldehyde in phosphate buffered saline (PBS) or 2.5% glutaraldehyde in PBS can be used for fixation. Glutaraldehyde should better fix proteins due to the presence of two aldehyde groups compared to formaldehyde. The staining is performed with a 0.5% solution of crystal violet in 20% methanol in PBS or water. It is also possible to quantitatively determinate proliferation. After adding 1% sodium dodecyl sulfate, crystal violet is released and the optical density is measured at 595 nm in a microplate reader [50].

The most advanced method in cell morphology monitoring is scanning electron microscopy (SEM), revealing morphological characteristics of cells, the ECM and minerals, which are the main components of mature bone tissue [51]. In the preparation of cells for SEM, dehydration by ascending alcohol series and critical point drying with hexamethyldisilazane are used, followed by drying in a desiccator [52]. However, monitoring of cell morphology itself to identify cells is not entirely reliable, because most cells change their appearance during culture growth; in addition, many cell types look very similar [51].

Fluorescent staining of specific cell structures can provide more detailed information. For good morphological evaluation of fluorescently stained cells, it is appropriate not to exceed 60% of their confluence. Cells can be counted and evaluated under a microscope for their spreading area, the cell perimeter and circularity; therefore, this staining is used for testing of cell adhesion to materials for bone tissue engineering [53].

Fluorescence staining of the cytoskeleton and cell nucleus is often used. The cytoskeleton, consisting of three types of filaments (actin microfilaments, intermediate filaments and microtubules), provides the cell with shape, mechanical stability and mobility and is also involved in cytoplasmic transport. Actin, involved in cell adhesion and spreading, allows formation of intercellular junctions and holds cells in the intercellular mass [54]. For staining filamentous polymerized actin (F-actin), the cells are fixed and permeabilized with 0.1% Triton X-100 in PBS. Then, blocking solution is applied and finally the cells are incubated in the premix of Phalloidin–Tetramethylrhodamine B isothiocyanate (TRITC) (Sigma-Aldrich, Saint Louis, MO, USA). After imaging the cells in a fluorescence microscope, the actin cytoskeleton is observed to be red [48].

More than 150 proteins, such as vinculin, talin and kindlin, have been identified in protein complexes called focal adhesions, which link integrin to the actin cytoskeleton and thus promote cell adhesion, migration and proliferation [55, 56]. Vinculin, a membrane–cytoskeletal focal adhesion protein, serves to anchor actin filaments to the membrane; therefore, it plays an important role in cell spreading, lamellipodia formation and cell shape control [57]. Mouse monoclonal anti-vinculin antibody conjugated to FITC (Fluorescein-5-isothiocyanate) (Sigma-Aldrich, Saint Louis, MO, USA) is used to visualize focal adhesions. In process of staining, it can be added to the premix at the same time as Phalloidin–TRITC, highlighting the focal adhesions in green.

It is also advisable to add nuclear staining, for example, using DAPI (4′,6-diamidino-2-phenylindole), which emits blue fluorescence when bound to DNA. There are commercially available ready-to-use kits as NucBlue™ Fixed ReadyProbes™ Reagent (Thermo Fisher Scientific, Waltham, MS, USA), which is directly added in an amount of approximately 2 drops per 1 mL of culture medium and blue fluorescence is observed after 15–30 min.

Cell viability and proliferation can be estimated by commercially available simple, rapid and sensitive kits. The Cell Counting Kit-8 (CCK-8, Bimake, Munich, Germany) is a ready-to-use solution that utilizes the highly water-soluble tetrazolium salt WST-8 [2-(2-methoxy-4-nitrophenyl)-3-(4-nitrophenyl)-5-(2,4-disulfophenyl)-2H-tetrazolium, monosodium salt] to produce a water-soluble formazan dye upon reduction by cellular dehydrogenases. The amount of dye generated by the activity of dehydrogenases in cells is directly proportional to the number of living cells. The absorbance is measured at 450 nm.

Another ready-to use kit is the PrestoBlue™ Cell Viability Reagent (Thermo Fisher Scientific, Waltham, MS, USA). This reagent also does not require cell lysis. It is resazurin-based solution for quantitatively measuring the viability and proliferation of cells. After addition to viable cells, the PrestoBlue™ is reduced, and turns red in color, becoming highly fluorescent. Fluorescence or absorbance can be detected. This is a sensitive method that provides results after only 10 min of incubation.

The RealTime-Glo™ MT Cell Viability Assay (Promega Corporation, Madison, WI, USA) is a nonlytic, homogeneous, bioluminescent method to measure cell viability in real time using a simple, plate-based technique. The reagent contains NanoLuc Luciferase and a modified substrate, which is reduced by metabolically active cells to NanoLuc Substrate. Upon its release into the medium, where it is rapidly used by the NanoLuc Enzyme, a bioluminescence proportional to the number of viable cells is generated.

### Determination of the mineralization degree and evaluation of osteodifferentiation

Osteogenic cells involved in the process of bone formation are responsible for production of insoluble inorganic components in the ECM containing calcium and phosphates (hydroxyapatite).

To determine the identity and quantity of mineral deposits in tissues, different methods are used. Von Kossa is a traditional histological staining technique based on a precipitation reaction of silver ions with phosphates under acidic conditions [58]. The yellow coloration in the early stage of the reaction indicates calcium phosphate. The photochemical conversion of silver phosphate to black metallic silver occurs after illumination. A limitation of this historical method is poor specificity. Positive staining can be observed not only due to hydroxyapatite deposits, but also due to mineralization of unknown origin [59].

Another method, alizarin staining, is used to confirm the presence of calcium [60]. Staining with alizarin identifies extracellular calcium deposition in the ECM of osteocytes in differentiated culture. For quantification, the dye is dissolved in cetylpyridinium chloride, and assessed by spectrophotometry [61]. A more sensitive but laborious method is the extraction of alizarin with acetic acid followed by neutralization with ammonium hydroxide and colorimetric detection at 405 nm. This method is used for weakly mineralized samples [62]. Another possibility for the quantification of calcium in cell culture is the use of commercially available kits, e.g., the Calcium Colorimetric Assay (Sigma-Aldrich, Saint Louis, MO, USA) which is based on spectrophotometric measurement of the product formed by the reaction between calcium ions and o-cresolphthalein [53, 63].

**Fig. 3:**
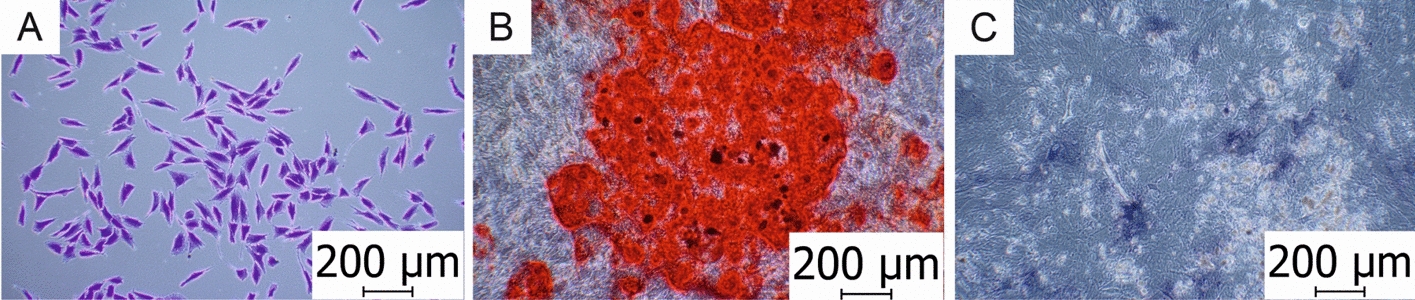
Cell staining. **A** Crystal violet staining assay used for morphological evaluation of human osteoblast-like MG-63 cells (MG-63). **B** Alizarin staining used for localization and quantification of calcium deposits of bone marrow-derived mesenchymal stem cells (BM-MSCs). **C** ALP used as an early osteogenic marker of bone calcification (BM-MSCs). Photographed on microscope Olympus CKX41 with a ×10 magnification lens. Scale = 200 μm

ALP activity can be detected by the BCIP®/NBT Liquid Substrate System (Sigma-Aldrich, Saint Louis, MO, USA) solution, which produces an insoluble blue to purple NBT diformazan product. The colour is very stable, and it does not fade after light exposure. After cell lysis, ALP can be quantified by measuring absorbance at 405 nm using a microplate reader [61]. This determination can be supported by the total protein content estimation. Examples of cell staining can be seen in Fig. 3.

The immunohistochemical staining is also used to visualize specific proteins structures, such as Col1 or OCN [55, 64].

### Gene expression profiling

With the introduction of real-time PCR (RT-qPCR) and other molecular biology techniques, such as next generation sequencing (NGS), there has been a big advance in the field of gene expression analysis. Real-time qPCR allows the detection of products formed during PCR by increasing the fluorescence intensity proportional to the amount of PCR products formed. Expression of specific genes can be used as a marker of differentiation. The list of genes suitable for osteodifferentiation assessment is shown in Table 1.

**Table 1:** Genes and primers for monitoring osteodifferentiation

Gene	Forward sequence	Reverse sequence
Glyceraldehyde-3-phosphate dehydrogenase (GAPDH)—housekeeping	GAGTCCACTGGCGTCTTCAC	GTTCACACCCATGACGAACA
Runt-related transcription factor 2 (Runx2)	GTAGATGGACCTCGGGAACC	GAGCTGGTCAGAACAAAC
Osterix (Osx, Sp7)	TTCTGCGGCAAGAGGTTCACTC	GTGTTTGCTCAGGTGGTCGCTT
Alkaline phosphatase (ALP)	GGAACTCCTGACCCTTGACC	TCCTGTTCAGCTCGTACTGC
Collagen type I (Col1)	GAGTGCTGTCCCGTCTGC	TTTCTTGTTCGGTGGGTG
Bone sialoprotein (BSP)	GGCAGTAGTGACTCATCCGAAG	GAAAGTGTGGTATTCTCAGCCTC
Osteopontin (OPN)	GTTTCTCAGACCTGACATCC	CATTCAACTCCTCGCTTTCC
Osteonectin (SPARC, BM-40)	TGCCTGATGAGACAGAGGTGGT	CTTCGGTTTCCTCTGCACCATC
Osteocalcin (OCN)	GGCAGCGAGGTAGTGAAGAG	CTCACACACCTCCCTCCTG
Fibroblast growth factor-23 (FGF-23)	GGAACAGCTACCACCTGCAGAT	CACCACAAAGCCAGCATCCTCT
Bone morphogenetic protein-2 (BMP2)	CAGACCACCGGTTGGAGA	CCACTCGTTTCTGGTAGTTCTTC
Receptor activator of nuclear factor κB ligand (RANKL)	GCCTTTCAAGGAGCTGTGCAAAA	GAGCAAAAGGCTGAGCTTCAAGC
Osteoprotegerin (OPG)	GGTCTCCTGCTAACTCAGAAAGG	CAGCAAACCTGAAGAATGCCTCC
Sclerostin (SOST)	GGAGCTGGAGAACAACAAGACC	TCACGTAGCGGGTGAAGTGCAG

According to ref. [65]

Another important technique used in molecular biology is Western blotting. This method is used to separate and identify proteins of interest. The protein mixture is separated by electrophoresis. These are then transferred to a membrane, which is incubated with a labeled specific antibody to the target protein. Bound antibodies are detected from visible bands on the membrane, where the thickness of the band corresponds to the amount of protein [66].

## Cells in bone tissue research

**Fig. 4:**
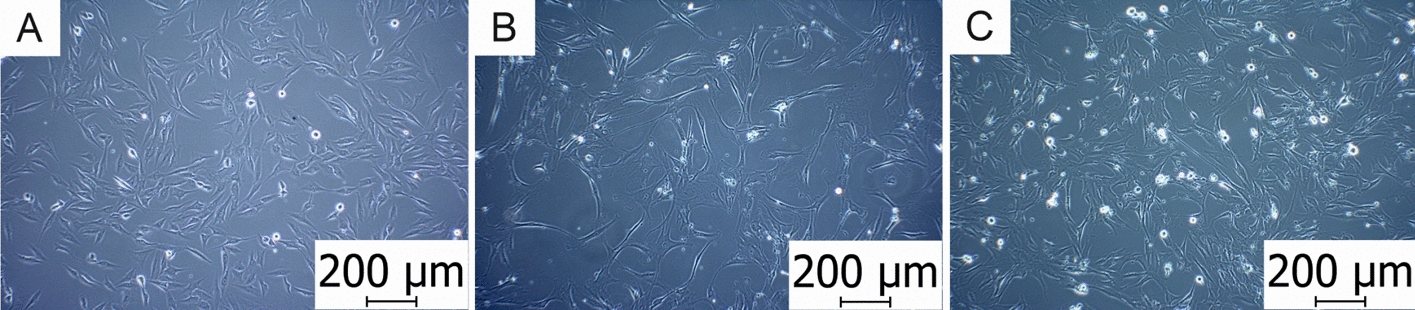
**A** Osteosarcoma cells (MG-63). **B** Mesenchymal stem cells (BM-MSCs). **C** Osteoblast cells (hFOB 1.19). Photographed on an Olympus CKX41 microscope with a ×10 magnification lens. Scale = 200 μm

Human cell cultures of various origins, from MSCs through primary cells to malignant cell lines, are used for in vitro testing of the material biocompatibility (Fig. 4).

### Human malignant cell lines

Cytocompatibility of biomaterials is often examined using osteosarcoma cell lines instead of primary osteoblasts due to their easier maintenance. Osteosarcoma cells are available in a number of cell lines that differ in their mutant gene(s). Their advantage is that a large amount of cells can be easily obtained, but the pathological phenotype could be a problem.

Many osteosarcoma-derived cell lines have characteristic features of osteoblasts, e.g., express specific receptors for vitamin D3 and PTH, exhibit ALP activity, and produce specific bone matrix proteins. However, the tumor origin of osteosarcoma cells can lead to significant phenotypic differences. These cells can differ from primary osteoblasts in their morphology, mitotic rate, expression profile of some cytokines, growth factors and matrix proteins, as well as in their mineralization activity [67]. Osteosarcoma cells also differ from osteoblasts in size. The mean size of attached osteosarcoma cells is approximately 1/6 of the size of osteoblasts. The population doubling time (PDT) of these cells is 2–3 times higher than that of osteoblasts [68, 69].

Genetic mutations involving numerous genes are associated with the onset and progression of osteosarcoma. Because of the different genetic backgrounds, their phenotypic variability must be taken into consideration in cytocompatibility studies. In the study of cellular and material interactions, it is not entirely clear to what extent malignant cell lines reproduce the behavior of primary osteoblasts [70].

There are a number of lines that can be used for various purposes, but mainly for cancer research. Examples can be seen in Table 2. The most commonly used osteosarcoma cell lines in bone tissue and cancer research, MG-63 and Saos-2, will be described in detail.

**Table 2:** Most used osteosarcoma cell lines

Osteosarcoma cells	Use in research	Refs.
MG-63	Testing of materials for use in bone tissue engineering Cancer treatment research	[2, 71]
Saos-2	Application in bone tissue engineering Research on cancer, metastatic problems, recurrence	[72, 73]
143B	Cell migration and proliferation research Research on useful substances for reducing proliferation and viability	[74–76]
HOS	Research on active substances that cause apoptosis and reduce viability	[77, 78]
U-2 OS	Testing of tomatidine cytotoxicity against cancer	[79–81]
143.98.2	Cytotoxicity testing of doxorubicin and cisplatin in the 2D culture and 3D scaffold	[82]
SJSA-1	Cytotoxic effect of lycorine stopping the cell cycle and causing apoptosis Development of a therapeutic agent targeting canonical Wnt/β-catenin	[81, 83]
MNNG/HOS	Research on oncogenes	[84]

#### MG-63

The human osteoblast-like MG-63 cell line (ATCC CRL-1427) (ATCC, Manassas, VA, USA) was derived from the osteosarcoma of a 14-year-old male. These cells of fibroblast spindle-shaped morphology are adherent and suitable for transfection. They are smaller than cells of human osteoblasts (hOB) [69].

MG-63 cells have the phenotype of immature osteoblasts. They are commonly used in in vitro studies to assess 3D scaffolds’ potential as bone tissue engineering materials [71] or to examine cytocompatibility of metallic implants with different surface treatment [8]. This line of cells has attracted a lot of attention, because it produces a large amount of interferon—a protein of non-specific immunity with an antiviral effect [85].

These cells show rapid cell growth without exhibiting contact inhibition, resulting in the formation of aggregates [86]. Their hormonal administration response is similar to hOB, but their disadvantages are arresting in a pre-osteoblast state and their low or variable cell mineralization. Inconsistencies appear in the literature regarding the mineralization capabilities of MG-63, but MG-63 cells are generally considered to exhibit low mineralization [87]. Calcium accumulation begins after day 28. ALP activity increases until day 15, then decreases to basal levels. Col1 expression is higher at days 7 and 15 than day 29. Expression of OCN and osteonectin has been observed between days 15 and 29, while Runx2, BSP and OPN were not detected [88]. Furthermore, it has been reported that the expression of angiogenic markers (VEGF, CD31, eNOS) increases significantly during differentiation [89]. MG-63 cells have a lower expression of Osx gene than Saos-2 and hOB cells. The behavior of the gene appears to be dependent on the maturation stage of the osteoblast phenotype. The response of MG-63 cells to vitamin D3 is similar to hOB. OCN is detected in supernatant from the cells only after treatment with vitamin D3 [68].

MG-63 cells exhibit features of cells poorly committed to the osteoblastic pathway, representing preosteoblastic or fibroblastic cells. Due to their rapid proliferation and easy cultivation, MG-63 cells serve well as an in vitro model cell line for initial cytocompatibility and adhesion assays. Nevertheless, with regard to their low calcium storage capacity and insufficient osteoblastic function, different proliferation rates, ALP activity and the ECM formation, they are not suitable for differentiation tests of biomaterial surfaces [89].

#### Saos-2

Human osteosarcoma cell line Saos-2 (ATCC HTB-85) (ATCC, Manassas, VA, USA) was isolated from an 11-year-old Caucasian female in 1975. These adherent polygonal cells with a mature osteoblast phenotype are smaller than MG-63, and have an epithelial cell morphology [90].

While MG-63 cells proliferate rapidly, Saos-2 are slower in proliferation, making them more similar to hOB. Osteosarcoma cell lines secrete lower levels of fibronectin into the medium than hOB. Saos-2 cells show the lowest level of FN. Immunofluorescence staining has revealed that hOB form an extensive network of FN fibrils between and across cells, while osteosarcoma cells produce only weak and short fibrils [70].

The expression of cytokines and growth factors in Saos-2 cells is similar to hOB. Saos-2 has been shown to express PTH and vitamin D3 receptors, which are similar to hOB in vitro and in vivo. In addition, dexamethasone increases their sensitivity to PTH, vitamin D3 and 17-β-estradiol [91].

The expression of genes controlling cell differentiation into osteoblasts, Runx2 and Osx, is similar to hOB in Saos-2 cells. The expression of these genes is mostly at undetectable levels in MG-63 cells. Staining for pluripotency markers STRO-1, Oct4 and Sox2 has revealed that both osteosarcoma cell lines (MG-63, Saos-2) remain undifferentiated [70, 92]. ALP activity in Saos-2 can be stimulated using dexamethasone and phosphate substrates in medium, which increases differentiation. Due to the much higher level of ALP activity in Saos-2, the use of dexamethasone should be considered. In the presence of β-glycerol phosphate, the cells strongly accumulate calcium and form a calcified matrix. The structure of collagen synthesized by Saos-2 cells is similar to collagen formed by hOB, but with a higher level of lysine hydroxylation [93].

In tests with a titanium material, the material’s surface inhibits the growth of Saos-2 and hOB, whereas this effect was not observed in MG-63 cells. However, MG-63 cells do not mimic the behavior of hOB as well as Saos-2 [70].

Although the Saos-2 line is of osteosarcoma origin, it retains important markers of osteogenic cell differentiation, particularly ALP activity and OCN expression at similar levels as hOB. Of the osteosarcoma cell lines, Saos-2 is, therefore, considered to be the most representative model of osteoblasts used to study cell-material interactions [53].

### Human mesenchymal stem cells (hMSCs)

Human mesenchymal stem cells (hMSCs) are multipotent adherent cells of the fibroblast type with the potential to differentiate into diverse cell types, such as osteoblasts, chondrocytes and adipocytes, under specific stimuli from culture media or biomaterials [94]. The hMSCs can be identified using the positive markers CD105, CD73 and CD90 (> 95%) and negative markers CD45, CD34, CD14 or CD11b, CD79alpha or CD19 and HLA-DR (< 2%), as defined by the International Society for Cellular Therapy (ISCT) [95].

These cells possess unique reparative abilities and they can be used in a wide range of cell-based treatments, particularly in connection with scaffolds. The use of hMSCs for testing of the biocompatibility of scaffolds and implants in vitro provides more relevant results than pre-differentiated and immortalized cell lines. It enables accurate evaluation of the influence of biomaterials on cellular activity. The use of hMSCs is also desirable, because scaffold technology is primarily intended for human medicine [3].

**Fig. 5:**
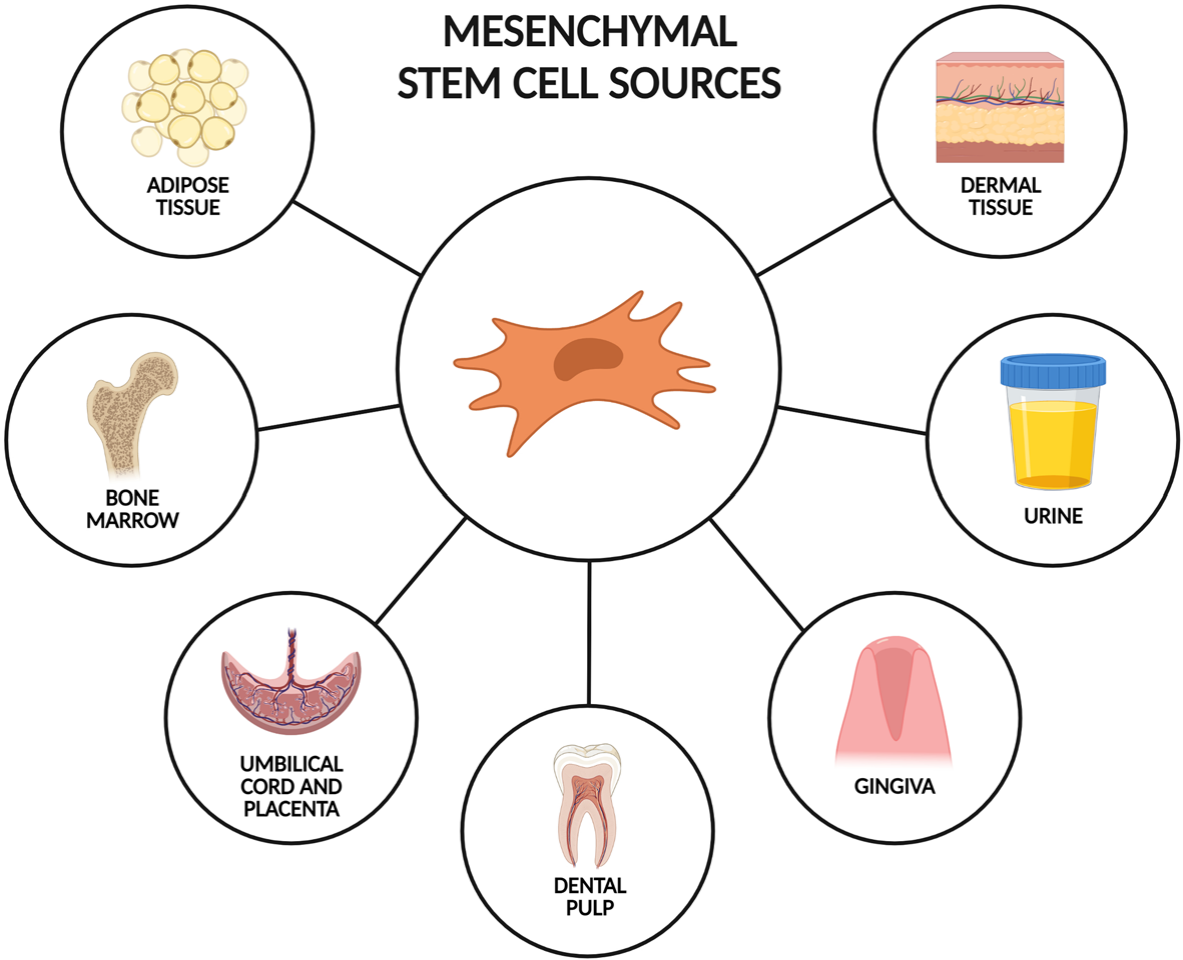
Most important sources of hMSCs. Created with BioRender.com

The most-studied sources of hMSCs include bone marrow, adipose tissue and umbilical cord blood. Recently, dental pulp stem cells, such as stem cells from human exfoliated deciduous teeth, gingival mesenchymal stem cells, urine-derived stem cells and dermal mesenchymal stem cells, have also been shown to have promising potential [96] (Fig. 5).

However, the yield of hMSCs from harvested tissue is usually very low. In the bone marrow hMSCs make up less than 1% of the cell fraction and in the adipose-derived stromal vascular fraction about 1.4%. This highlights the need to achieve an increased number of hMSCs by in vitro cultivation, so that the required number of cells can be obtained for implantation or research [61]. To achieve osteogenic differentiation, osteogenic supplements such as β-glycerolphosphate, ascorbic acid, dexamethasone [97] and FGFs are most often added to the growth medium [89].

The hMSCs according to origin are discussed in detail in the following text.

#### Adipose-derived mesenchymal stem cells (AD-MSCs)

Human adipose tissue may be considered the most appropriate alternative source of MSCs. A large number of studies show that adipose tissue contains a biologically and clinically interesting heterogeneous cell population called the stromal vascular fraction [98]. AD-MSCs can be extracted from subcutaneous human adipose tissue during elective liposuction [99].

The purity of the isolated AD-MSCs is assessed through flow cytometry analysis. A homogeneous cell population contains cells positive for CD105, CD44, CD90, CD73 and negative for CD45 surface markers [97].

This cell type has similar phenotypic and functional characteristics to bone marrow-derived mesenchymal stem cells (BM-MSCs) [98]. Both AD-MSCs and BM-MSCs have fibroblast-like morphology and express similar surface markers. However, it remains unclear whether they are equivalent to MSCs derived from other tissues. For example, the surface marker CD44 is more highly expressed by BM-MSCs, which can lead to different biological properties between AD-MSCs and BM-MSCs. Surface markers should be determined in freshly isolated cells, as their expression may change during cultivation. The choice of cells depends on experimental use. By selection based on a specific marker, a defined population can be isolated [100]. For example, if the fraction is selected on the basis of the CD271 + marker, there is the greatest potential for differentiation into osteoblasts.

The use of AD-MSCs to evaluate the biocompatibility of scaffolds as an osteogenic platform is valuable for therapeutic applications [3]. In addition, the advantage of AD-MSCs cells over BM-MSCs is the possibility to harvest them in a less invasive manner [98].

#### Bone marrow mesenchymal stem cells (BM-MSCs)

BM-MSCs are autologous adherent cells of fibroblast morphology which are involved in reparation of damaged bones. During bone injury they migrate to the damaged site, where they differentiate into osteoblasts, and secrete bioactive factors that promote bone repair [101].

Their main source is the bone marrow, but unfortunately, the number of harvested cells and their differentiation potential decreases rapidly with age of the donor [96]. They are obtained either by isolation from bone marrow taken by an invasive method, i.e., by puncture from the hip ridge [102], or in a non-invasive manner, i.e., by separation from the blood after previous drug stimulation or during routine orthopedic surgery from donors [89].

Despite the lack of a single specific surface marker, panels of markers to help identify BM-MSCs have been proposed. However, recent experiments show that these cells are not a single phenotypic population. In the study of Walter et al. BM-MSCs show a different phenotype according to the isolation technique, even from the same donor and same site of harvesting. While cells from outgrowth cultures express higher levels of CD10, CD49f and CD56, cells from aspirate cultures are associated with higher expression of CD146 [103].

The primary culture of BM-MSCc becomes homogenous after 2–3 passages, then enters into senescence very soon during cultivation, losing the properties of stem cells. After 8–10 passages they show abnormalities typical of the Hayflick´s model of cell aging, which is manifested by morphological abnormalities, cell enlargement, and finally termination of proliferation. The stability of mesenchymal cell markers is decreased starting in passage 7, which may be related to cell population heterogeneity and senescence during long-term cultivation in vitro [104].

The PDT of AD-MSCs and BM-MSCs is very different up to passage 2; this takes about 80 h for BM-MSCs, while it is about 50 h for AD-MSCs. Between passages 2 and 6, the PDT is nearly the same (between 30 and 35 h). The PDT increases again starting in passage 7 and it can increase up to 160–180 h for BM-MSCs in long-term cultures [105].

BM-MSCs differentiate into bone cells more efficiently than AD-MSCs. Ye et al. reported higher mineralization and ALP activity in BM-MSCs culture compared to AD-MSCs [106]. This result was confirmed by other studies, where BM-MSCs had a higher potential for osteogenesis and chondrogenesis than AD-MSCs [107]. It was found that hypoxia enhances osteogenesis and suppresses adipogenesis. The expression of osteogenesis-related genes, such as ALP, Col1 and OCN, was significantly increased under hypoxia. This corresponds to the conditions of the unique microenvironment in the bone marrow niches, where the mesenchymal cells reside [108].

After adding osteogenic supplements to the medium, BM-MSCs differentiate well into osteoblasts. However, immortalized BM-MSCs have been found to have a potential for neoplastic transformation, so their use in therapy can be risky [109].

#### Human umbilical cord-derived mesenchymal stem cells (HUC–MSCs)

HUC–MSCs are obtained non-invasively from various parts of the umbilical cord, i.e., Wharton's jelly, cord lining, and the perivascular region, with Wharton's jelly being the most widely used. The enzymatic cleavage method provides a more homogeneous cell population and a larger number of cells, but still provides a lower concentration of MSCs than in bone marrow or adipose tissue [110].

Morphologically, HUC–MSCs resemble stem cells obtained from bone marrow or adipose tissue [97]. Their surface markers are similar to mesenchymal cells, except for the negativity for CD133. However, their phenotype may be affected by the isolation method (explant method or enzymatic method), medium used, and number of passages. HUC–MSCs are primitive stem cells, intermediate between embryonic stem cells and adult mature cells, as evidenced by their low expression of pluripotency markers (Oct4, Nanog, Sox2 and KLF4) [111].

In the presence of indomethacin, these cells are able to differentiate into adipocytes. Their chondrogenic differentiation potential is reported to be up to 3 times higher than that of BM-MSCs. They can also differentiate into neurons, astrocytes, and glial cells and produce neuronal proteins [110]. HUC–MSCs have been shown to express liver markers and can differentiate into hepatocytes [112]. Another study showed that HUC–MSCs could be also induced to differentiate into insulin-producing cells of Langerhans islets [113].

HUC–MSCs are still used mainly in regenerative medicine for liver cell therapies, in the treatment of autism, type 1 and type 2 diabetes, stroke, spinal cord and brain injuries, Parkinson's and Alzheimer's disease, and multiple sclerosis. Clinical studies are currently investigating their potential anti-cancer effects [110]. An in vivo study showed that 3-week intravenous injections of HUC–MSCs may impair breast or lung tumor growth. Thus, they may be a useful tool for cancer cytotherapy [114].

As far as osteogenic differentiation, HUC–MSCs differentiate into osteoblasts insufficiently. Although HUC–MSCs have a significantly higher proliferation capacity than BM-MSCs and maintain high activity after multiple passages, they form a less mineralized ECM, which suggests a somewhat lower osteogenic capacity than BM-MSCs [94]. The osteogenic properties of HUC–MSCs can be significantly improved by the use of osteoinductive growth factors, such as BMP2 and BMP7, so the expression levels of osteogenic genes are comparable to those of BM-MSCs [115]. Research in biomaterials has shown that some hydrogels may also promote osteodifferentiation of HUC–MSCs. HUC–MSCs can be harvested non-invasively and have favorable proliferation capacity, low immunogenicity and a strong immunosuppressive capacity. These properties make them promising for bone tissue engineering [94].

#### Dental pulp stem cells (DPSCs)

Teeth are at risk of damage caused by mechanical trauma, chemicals, cancer or bacterial infections. Unlike bone, which can be remodeled and repaired, teeth show limited reparative processes and do not easily regenerate completely. After crown formation, programmed ameloblast cell death and loss of enamel repair ability occur [116].

The dental pulp in the pulp cavity of the tooth contains connective tissue, neural fibers, blood and lymphatic vessels, and DPSCs. Its main function is formation and physiological maintenance of dentin [116]. DPSCs play a significant role in maintaining marrow homeostasis and repairing injuries, and can differentiate into odontoblast-like cells and protect the underlying marrow by forming new dentin [117, 118].

DPSCs have been found in multiple stem cell niches, which are localized in capillaries and nerve networks in the cell-free zone, in the innermost pulp layer in the cell-rich zone and in the outermost layer, which contains odontoblasts [119]. In these niches, there are specific interactions between the DPSCs and their surrounding microenvironment. DPSCs are functionally regulated by the ECM, other local cell types and bioactive molecules that influence the fate of cells (self-renewal or differentiation) [117, 120].

DPSCs can be obtained after extraction from a child´s primary deciduous teeth or healthy wisdom teeth; there are a large number of them in the dental pulp and they have a greater ability to proliferate than other stem cells, such as BM-MSCs or AD-MSCs [119, 121, 122]. DPSCs isolated in this way are characterized as highly clonogenic with multidifferentiation and neurovascular properties [118]. DPSCs were found to have a faster PDT compared to BM-MSCs and AD-MSCs [121].

The dental pulp stem cell immunophenotype contains MSC markers, such as CD73, CD90, and CD105. It was further found that another MSC marker, STRO-1, was co-expressed with CD146 and pericyte antigen 3G5 and forms a specific niche in the dental pulp. In addition, DPSCs express neural lineage markers [122].

DPSCs have a high differentiation capacity, such as in neurogenesis, osteogenesis, chondrogenesis, angiogenesis and dentinogenesis. The differentiation process of DPSCs is affected by growth factors, e.g., FGFs, transforming growth factor-β (TGF-β), nerve growth factor, platelet-derived growth factor and BMPs [122]. Osteogenic supplements are added into the medium to induce differentiation of DPSCs into osteoblasts. Osteodifferentiation was demonstrated by an increase in ALP levels and staining of deposited calcium with alizarin [121]. However, compared to BM-MSCs, DPSCs produce only sporadic calcified nodules, and when they were transplanted into immunocompromised mice, they created a dentin-like structure lined with odontoblast-like cells. DPSCs and BM-MSCs show similar expression of ALP, Col1, osteonectin, OPN, OCN and FGFs. DPSCs have no expression of BSP, while in BM-MSCs, it is present, but at low levels [123].

DPSCs are currently being tested as a possible root canal filling, instead of the inert materials, which result in a reduction of dental vitality and sensitivity [124]. These cells are also being tested for their adhesion, proliferation and osteodifferentiation on surfaces of dental implants [121]. Recently, these cells have also received attention due to their ability to secrete neurotrophic factors effective for axonal survival and regeneration, making them an alternative source for peripheral nervous system regeneration therapy [125].

#### Gingival mesenchymal stem cells (G-MSCs)

G-MSCs represent a subpopulation of spindle-shaped fibroblast-like cells and maintain this morphology in late passages. They were first isolated in 2009 by Zhang et al. [126]. G-MSCs have a high level of self-renewal, differentiation and immunomodulation demonstrated both in vitro and in vivo. Their collection is simple. The cells can be easily isolated from the gingival tissue discarded directly during routine surgery or a surgical biopsy [127]. Previously, tissue from gums affected by periodontitis was considered biological waste and was removed during surgery [96]. However, even from a small gum biopsy, a large number of MSCs in primary culture can be obtained in a short time [104].

In patients suffering from periodontitis with jawbone resorption, a classic autologous graft is used when a dental implant is needed, because osteointegration is only possible if there is sufficient bone volume available to place the dental implants and form a firm connection. However, this operation is an expensive procedure with a risk of complications, so bone tissue engineering using MSCs, biomaterials and active biomolecules is a great alternative [96].

Primary cultures of G-MSCs are homogeneous populations showing a stable morphology, phenotype, karyotype, normal telomerase activity, and stability of MSCs markers even in long-term cultures [127]. They are negative for the hematopoietic markers CD14 and CD34, weakly positive for CD45, and highly positive for the MSC markers CD73, CD90 and CD105 [128].

The PDT of G-MSCs remains the same, in the range of 30–50 h, from primary to long-term cultures. In an effort to increase the number of cells in in vitro cultivation, G-MSC cell growth is approximately threefold higher than that of BM-MSCs [104].

After adding specific supplements to the medium, G-MSCs can differentiate into mature osteoblasts, chondrocytes, myocytes and adipocytes [129], and also into epithelial and nerve cells [130]. In the presence of osteogenic supplements in the medium, G-MSCs form mineralized nodules and show expression of osteoblast-specific genes [131]. When comparing the behavior of G-MSCs and BM-MSCs on a scaffold in vitro, staining against OCN showed strong expression in G-MSCs, indicating their potential for in vivo bone regeneration. The osteogenic differentiation potential of G-MSCs is lower than BM-MSCs; however, it can be increased by treatment with TGF-β [132]. Due to their uncomplicated collection and faster proliferation, G-MSCs seem to be a good alternative for applications in cell therapies and for bone regeneration associated with the scaffolds [130]. In addition, G-MSCs are not tumorigenic, so their in vivo therapeutic use is potentially safe [133]. The regenerative potential of G-MSCs may also be affected by factors, such as the donor´s health, age and lifestyle, which must be taken into account [134].

#### Urine-derived stem cells (USCs)

The presence of cells in urine that display stem cell properties was described in 2008 [135]. These cells originate from the pericytes of kidney and urinary epithelium. However, this difference in the origin of cells can lead to the presence of different cell populations in the urine [136].

Currently, USCs are of interest due to their features and differentiation capacity. Their simple, safe and inexpensive isolation from excreted urine is a huge advantage over the invasive collection of most types of MSCs. Furthermore, these cells can also be isolated by biopsies from renal pelvis, ureter, bladder and urethra. Although a larger number of the cells can be obtained with this method, it is better to avoid this invasive procedure [137].

The urine contains about 1 to 2 cells per 100 mL, which can be attached to culture dishes and proliferate [135]. Freshly isolated cells have two types of shapes: spindle-shaped and rice-shaped. By gradual passage, the cells acquire elongated spindle-shaped fibroblast-like morphology. The PDT differs between passages, from about 20 h in P1 to about 28 h in P5 [138].

USCs are positive for the MSC markers CD44, CD73, CD90, CD105, Vimentin and Col1 and negative for the hematopoietic markers CD14, CD34, CD117 and CD133 [139].

USCs have similar biological characteristics to AD-MSCs, but they have a higher proliferative capacity, which makes them an interesting alternative cell source [140]. Compared to BM-MSCs, USCs have better adipogenic and endothelial potential, as well as the ability to initiate new vascularization [141].

These cells can be differentiated into osteogenic, chondrogenic and adipogenic lines. The osteogenic differentiation of USCs can be further promoted by silver nanoparticles [142].

Other reports have described the differentiation of USCs into urothelial cells, smooth myogenic cells, endothelial cells, neuron-like cells and skeletal myogenic cells [143–146]. Differentiation into renal cells, podocytes and tubular epithelial cells is of high interest for reconstructive surgery in the genitourinary tract [138]. These cells can also find application in the diagnosis of genetic disorders, new drug testing and toxicology [137]. Their application for cartilage regeneration is also being tested; injection of hyaluronic acid containing USCs supported new formation of cartilage-like tissue in rabbits [147].

USCs can also be used in bone tissue engineering. It has been shown that they form calcium deposits and express ALP, OCN, OPN and BMPs. Due to these osteogenic features, they have potential for bone regeneration [148]. In experiments with rats, USCs were compatible with bone scaffolds and may be beneficial in repairing critical sized segmental bone defects [149, 150].

#### Dermal mesenchymal stem cells (D-MSCs)

Multiple populations of stem cells, which reside in the hair follicle, sebaceous gland, dermis and epidermis, have been found in the skin. Multiple types of stem cells may be isolated simultaneously from one skin sample, for example, epidermal stem cells, skin-derived precursors (SKPs) and dermal mesenchymal stem cells (D-MSCs) [151]. These cell types do not show any obvious differences from each other [152]. They probably differ in having higher gene expression of Nanog and Oct4 in SKPs, suggesting that SKPs have a larger differentiation potential [151].

D-MSCs have a fibroblastic spindle-shape morphology. They are positive for CD44, CD73, CD90 and negative for CD34, and their PDT is about 27 h. These cells have the capacity to differentiate into various mesenchymal lineages, such as osteoblasts, adipocytes, chondrocytes or myocytes [153]. Osteogenic differentiation has been tested by von Kossa staining, which showed calcified deposits. D-MSCs have been found to express osteogenic markers, such as ALP and OPN [154]. Osteodifferentiation was also demonstrated by staining with alizarin [155]. Research suggests that D-MSCs from hair follicle and BM-MSCs have similar features [153].

D-MSCs and SKPs have therapeutic potential in cell therapy and regenerative medicine and it is advantageous due to the easy availability of the skin, because it is the body´s largest organ and has high regenerative capacity [151]. Currently, D-MSCs are more often used for cartilage engineering than in the bone engineering [156].

### Human osteoblasts

Osteoblasts are cells of mesenchymal origin, producing osteoid components important for bone matrix formation [157]. The main advantage of using primary osteoblasts is their clinical applicability and the lack of need to address the interspecies differences seen in culture staining for ALP and calcium deposits, as shown in MC3T3-E1 mouse preosteoblasts and bovine and ovine osteoblasts. The disadvantage is their limited availability and phenotypic heterogeneity. There are two basic ways of harvesting osteoblasts—the explant method and the enzymatic method. The method of isolation influences the subsequent in vitro culture [87]. Models based on primary osteoblasts provide highly sensitive responses to material cytocompatibility assessment.

#### Human fetal osteoblast 1.19 (hFOB 1.19)

The cell line hFOB 1.19 (ATCC CRL-11372) (ATCC, Manassas, VA, USA) was established by transfection of fetal limb tissue after spontaneous miscarriage. Primary cultures isolated from fetal tissue were transfected with a gene coding for a temperature-sensitive mutant (tsA58) of SV40 large T antigen along with a gene coding for neomycin (G418) resistance [158]. This cell line is considered to be a preosteoblastic or osteoprogenitor cell line that has the potential to differentiate into an adipocytic and chondrogenic phenotype [159].

Cells grown at a permissive temperature of 33.5 °C exhibit rapid cell proliferation (PDT 36 h), whereas at a restrictive temperature of 39.5 °C the cell proliferation decreases rapidly (PDT 96 h). At this higher temperature, differentiation increases, the levels of ALP and OCN are higher and a mature osteoblast phenotype is formed [160].

Subramaniam et al. characterized hFOB 1.19 as an immortalized but untransformed cell line with minimal karyotype damage despite multiple passages [161]. In basic and applied research, immortalized cell lines are used more often than primary cells, because experiments with them are easier to repeat and more accessible. However, it is clear that immortalized cell models do not fully resemble primary cells [162].

The hFOB 1.19 cells express many osteoblastic markers, and they have high ALP activity, high OCN expression and cyclic adenosine monophosphate production. They have also been shown to spontaneously mineralize the ECM forming calcified nodules, and express low levels of estrogen receptors, [163], so they are also used to study the responses of human osteoblasts to hormones [161].

The hFOB 1.19 cells provide a homogenous, rapidly proliferating model system for studying normal human osteoblast differentiation, osteoblast physiology, and hormonal, growth factor, and other cytokine effects on osteoblast function and differentiation [164].

#### Human osteoblasts (hOBs)

The hOBs are post-proliferative cells, cuboidally shaped, with strong ALP expression and active bone matrix production [157]. The main components of the mineralized ECM are Col1, hydroxyapatite, growth factors, such as BMPs and TGF-β, with smaller but significant amounts of OCN, OPN, BSP and matrix GLA protein [165].

As a tissue model, hOBs have a physiological osteoblast phenotype. Compared to tumor or immortalized cell lines, hOBs are a model with the same genetic background mimicking the physiological environment. They are used to study the mechanism of bone formation, regulation of differentiation, and molecular and biochemical mechanisms associated with disease development; they are also used to monitor potential therapeutic agents, or to test the biocompatibility of bone replacements. As an in vitro model, hOBs are irreplaceable and should not be neglected during clinical trials [157].

The fact that they are human cells eliminates differences in cell behavior under experimental conditions that would occur when using cell lines from different animal models [166, 167]. On the other hand, the human source of cells can be considered limited. Human osteoblasts are mostly derived from the crest of the femur, knee bones and ribs. Primary cell cultures are then isolated from bone trabeculae using collagenase [168]. However, subsequent in vitro culture with fetal bovine serum or other growth factors can change gene expression and the cell phenotype of hOB [169]. The usefulness of the primary cell line lasts only several days, because long-term cultivation leads to phenotypic drift. Another negative aspect of primary cultures of these cells is that they undergo rapid senescence with limited regeneration and differentiation abilities [170].hOBs culture is also dependent on the collection site [171] and is affected by the health condition of the cell donor [170]. The cells may exhibit inter-individual variability and differences between the sexes [172, 173], because the stimulatory effect of 17β-estradiol on differentiation [174]. Some changes can also occur in epigenetic regulation, which can affect the results of study or therapy using hOBs [162].

In 3D cultivations, which are intended to mimic natural tissue and specific features of the human organ microenvironment, hOBs naturally differentiate to osteocytes [175]. However, compared to osteosarcoma cell lines, slow proliferation, worse availability, and time-consuming isolation are limiting factors for the use of hOBs in research [162].

## Advantages and disadvantages of selected cells in bone tissue research

The use of each cell model has its pros and cons, which should be considered according to the specific type of research. Table 3 should help with the selection of the appropriate cells for a particular application.

**Table 3:** Advantages and disadvantages of using different human cells in bone tissue engineering research

Cells	Use in bone tissue engineering	Advantages	Disadvantages
*Osteosarcoma cells*			
MG-63	Initial adhesion and biocompatibility assay	Fast growth and easy cultivation	Pathological phenotype; low mineralization
Saos-2	Initial adhesion and biocompatibility and osteodifferentiation tests	Fast growth and easy cultivation; a valuable pilot model due to high ALP activity and OCN expression	Pathological phenotype
*Mesenchymal stem cells*			
AD-MSCs	Cytocompatibility tests; usable for therapeutic applications	Relevant results; easier to collect than BM-MSCs	Lower differentiation potential for osteogenesis than BM-MSCs
BM-MSCs	Cytocompatibility tests; usable for therapeutic applications	Relevant results; high differentiation potential for osteogenesis	Invasive method of collection; high rate of senescence depending on the age of the donor; long PDT; tumorigenic potential of immortalized BM-MSCs; ethics committee approval and patient's informed consent needed to access primary cells
HUC–MSCs	Better for regenerative medicine and therapies of the nervous system, liver and diabetes	Non-invasive method of collection; favorable proliferation capacity; low immunogenicity	Delayed and insufficient osteogenesis
DPSCs	Testing of dental implants; peripheral nervous system regeneration therapy	Easy collection from deciduous teeth and wisdom teeth; faster PDT compared to BM-MSCs and AD-MSCs; wide differentiation potential	Weak calcification; differentiation mainly into odontoblasts
G-MSCs	Osteointegration of dental implants; testing of scaffolds for bone regeneration; application in regenerative dentistry	Easy collection from gum; faster PDT compared to BM-MSCs and AD-MSCs; for clinical applications better than BM-MSCs; no tumorigenic potential	Reduced osteogenic differentiation potential compared to BM-MSC
USCs	Possibility of use for cartilage and bone regeneration is in the research phase	Easy, safe and cheap collection from urine	Use in bone engineering is not yet common; rather for genitourinary tract reconstructive surgery
D-MSCs	More often for use in cartilage engineering (osteoarthritis treatment)	Easy availability of the skin with high regenerative capacity	Use in bone engineering is not yet common
*Osteoblasts*			
hFOB 1.19	Model for the study of cytokines and growth factors effect on osteoblast physiology and differentiation	Easier to repeat experiments than with hOBs; spontaneous differentiation	Transfected cell line
hOBs	Model for studying the mechanism of bone formation, regulation of differentiation, molecular and biochemical mechanisms associated with disease development, to monitor potential therapeutic agents, or to test the biocompatibility of bone replacements	Physiological phenotype of osteoblast differentiating into osteocyte	Limited resources of hOBs; long-term cultivation leads to phenotypic drift; high rate of senescence; donor age, gender and health dependent culture; ethics committee approval and patient's informed consent needed to access primary cells

## Conclusions

Bone regeneration after injury or after surgical bone removal due to diseases such as cancer is a major medical challenge. Regenerative strategies require cells capable of proliferation and differentiation in the bone niche. A variety of materials are currently being tested that could serve as a replacements for a missing bone or a tooth. It is difficult to find a universal human cell line to be used as a good model for all phases of scaffold or implant biocompatibility testing, because each has its advantages and disadvantages.

Osteosarcoma cells are preferred for initial adhesion assays due to their easy cultivation and fast proliferation. However, they are not suitable for subsequent differentiation testing due to their origin as cancer cells, and differences in genetic background resulting in abnormal physiological characteristics. MSCs are more suitable for biocompatibility testing, but they proliferate more slowly and soon undergo senescence, and some may show weak osteodifferentiation. The use of native hOBs provides relevant results in evaluating the effect of biomaterials on cellular activity and it is also desirable, because the production of these materials is primarily intended for human medicine. However, osteoblast resources are limited and these cells rapidly change their phenotype during culturing.

Selection of a suitable cell line plays a crucial role in the biocompatibility testing before clinical use of materials. The overview of cell models provided by this article can help in choosing the most appropriate human cells to use for a particular application in bone tissue engineering research.

## Data Availability

Not applicable.

## Abbreviations

AD-MSCs: Adipose-derived mesenchymal stem cells
ALP: Alkaline phosphatase
BM-MSCs: Bone marrow-derived mesenchymal stem cells
BMP: Bone morphogenetic protein
BSP: Bone sialoprotein
Col1: Collagen type I
D-MSCs: Dermal mesenchymal stem cells
DPSCs: Dental pulp stem cells
ECM: Extracellular matrix
FGF: Fibroblast growth factor
FITC: Fluorescein-5-isothiocyanate
G-MSCs: Gingival mesenchymal stem cells
hFOB 1.19: Human fetal osteoblast 1.19
hOBs: Human osteoblasts
HSC: Hematopoietic stem cell
HUC-MSCs: Human umbilical cord-derived mesenchymal stem cells
MSCs: Mesenchymal stem cells
OCN: Osteocalcin
OPG: Osteoprotegerin
OPN: Osteopontin
Osx, Sp7: Osterix
PBS: Phosphate buffered saline
PDT: Population doubling time
PTH: Parathyroid hormone
RANKL: Receptor activator of nuclear factor κB ligand
Runx2: Runt-related transcription factor 2
SEM: Scanning electron microscopy
SKPs: Skin-derived precursors
SOST: Sclerostin
SPARC, BM-40: Osteonectin
TGF-β: Transforming growth factor-β
TRITC: Tetramethylrhodamine B isothiocyanate
USCs: Urine-derived stem cells
